# Usefulness of detective flow imaging endoscopic ultrasound for pancreatic neuroendocrine tumors difficult to detect with other imaging modalities

**DOI:** 10.1111/den.14979

**Published:** 2024-12-10

**Authors:** Takaoki Hayakawa, Eisuke Iwasaki, Takanori Kanai

**Affiliations:** ^1^ Division of Gastroenterology and Hepatology, Department of Internal Medicine Keio University School of Medicine Tokyo Japan

## Abstract

Watch a video of this article.

## BRIEF EXPLANATION

Recently, detective flow imaging endoscopic ultrasound (DFI‐EUS), which can visualize microvascular blood flow, has been developed and integrated into an ultrasound observation system (ARIETTA 850; Fujifilm Healthcare, Tokyo, Japan). DFI‐EUS is characterized by its ease of application in routine observations, offering the advantage of minimal patient discomfort and eliminating the need for drug administration, unlike contrast‐enhanced EUS. However, the usefulness of DFI‐EUS for pancreatic neuroendocrine tumor (pNET) has rarely been reported.

**Figure 1:**
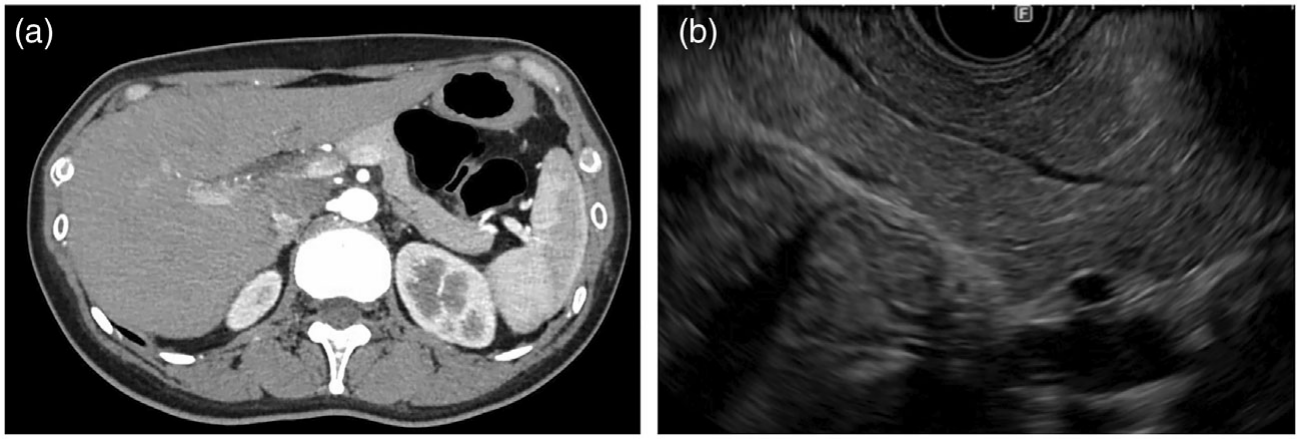
(a) Dynamic computed tomography could not detect the tumor. (b) The tumor could not be detected by B‐mode endoscopic ultrasound.

**Figure 2:**
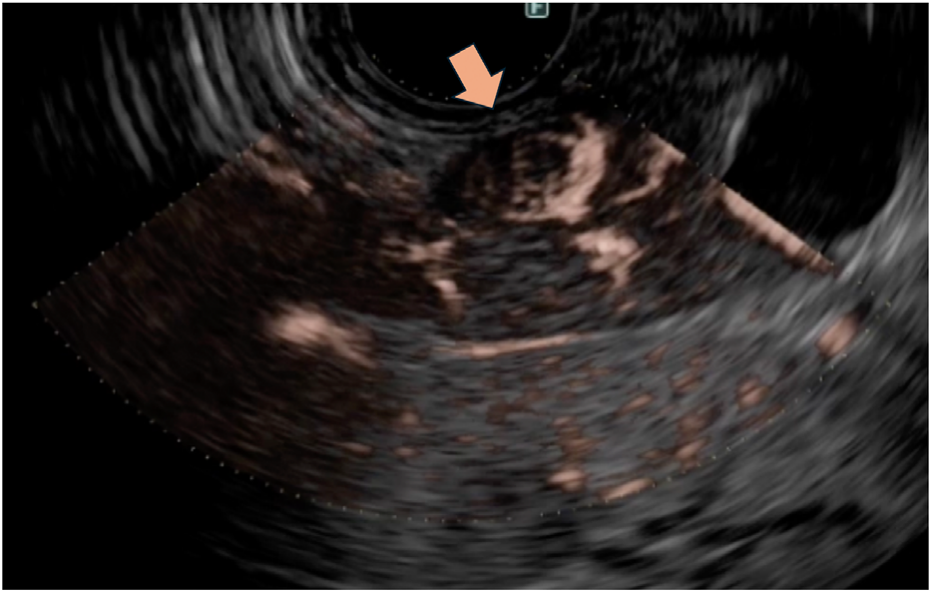
Detective flow imaging endoscopic ultrasound was able to visualize a small 8.9 mm tumor in the pancreatic tail (arrow) by depicting tumor vessels characteristic of pancreatic neuroendocrine tumors.

A 43‐year‐old woman with a history of hypoglycemic attacks visited our hospital. Dynamic computed tomography (CT) and magnetic resonance imaging (MRI), performed for further investigation, showed no abnormalities (Fig. 1a). However, based on the results of a glucose load test and selective arterial calcium injection test, an insulinoma located in the tail of the pancreas was suspected. EUS was conducted to identify the tumor's location and determine the extent of resection for surgery. The tumor could not be detected by B‐mode or conventional color Doppler EUS (eFlow‐EUS) (Fig. 1b), but only DFI‐EUS could visualize the tumor location by depicting blood vessels wrapping around the tumor from the periphery, allowing visualization of a small 8.9 mm tumor in the tail of the pancreas (Fig. 2). Surgery was performed later, and the diagnosis of insulinoma was confirmed (Video S1).

Although there has been a previous report of a pNET case observed using both B‐mode EUS and DFI‐EUS,^1^ this is the first reported case in which the tumor was detectable only with DFI‐EUS. It has been reported that pNETs are characterized by their hypervascularity,^2^, ^3^ and by visualizing this feature with DFI‐EUS, we were able to identify its location. When a pNET is clinically suspected but not detectable by other imaging modalities, DFI‐EUS may be valuable for detecting the tumor.

## CONFLICT OF INTEREST

Author E.I. serves as an Editor of *Digestive* *Endoscopy*. The other author declares no conflict of interest for this article.

## Supporting information


**Video S1** Detective flow imaging endoscopic ultrasound for pancreatic neuroendocrine tumor that cannot be detected by other imaging modalities.
